# Identifying Risk Factors and Constructing Predictive Models for Wearing‐Off and Dyskinesia in Chinese Patients With Parkinson's Disease on Long‐Term Levodopa Therapy

**DOI:** 10.1111/cns.70544

**Published:** 2025-08-11

**Authors:** Jing Zhao, Yunlei Gao, Chong Shi, Jia Chen, Yanhong Wang, Jiaqi Chen, Shaochen Ma, Peifu Wang, Jilai Li, Jichen Du, Zhirong Wan

**Affiliations:** ^1^ Department of Neurology Aerospace Center Hospital Beijing People's Republic of China; ^2^ Department of Geriatrics Aerospace Center Hospital Beijing People's Republic of China; ^3^ Department of Traditional Chinese Medicine Aerospace Center Hospital Beijing People's Republic of China

**Keywords:** dyskinesia, levodopa, motor complications, parkinson's disease, risk factors, wearing‐off phenomenon

## Abstract

**Aims:**

This study aimed to investigate the incidence and risk factors of motor complications including wearing‐off (WO) and dyskinesia during long‐term levodopa (LD) therapy in Chinese patients with Parkinson's disease (PD), and develop corresponding predictive models, thereby providing a basis for personalized treatment strategies.

**Methods:**

This cross‐sectional study included 208 consecutive PD patients who were recruited. The presence of WO and dyskinesia was assessed by a 9‐item wearing‐off questionnaire and the Unified Parkinson's Disease Rating Scale part IV. Univariate and multivariate logistic regression analyses were used to predict the risk factors of WO and dyskinesia. Predictive models for WO and dyskinesia were then constructed, and their diagnostic performance was evaluated using the area under the curve (AUC).

**Results:**

The overall prevalence rate of motor complications was 46.2% (96/208), with a prevalence of 45.7% (95/208) for WO, 22.1% (46/208) for dyskinesia, and 21.6% (45/208) for the simultaneous occurrence of WO and dyskinesia. Younger age at onset (OR 0.92, *p* < 0.001), higher levodopa‐equivalent daily dose (LEDD) (OR 1.00, *p* < 0.001), and higher Hoehn‐Yahr stage (OR 3.41, *p* < 0.001) were independent risk factors for WO. A predictive model for WO constructed using these three variables demonstrated high diagnostic efficacy with an AUC of 0.887 (95% CI 0.842–0.932), a sensitivity of 84%, and a specificity of 83%. The independent risk factors for dyskinesia included younger age at onset (OR 0.94, *p* < 0.001), akinetic‐rigid type (OR 2.42, *p* = 0.034), and higher LEDD (OR 1.01, *p* < 0.001). A predictive model for dyskinesia constructed using these three variables yielded an AUC value of 0.829 (95% CI 0.767–0.897), with a sensitivity of 67% and a specificity of 89%. The two models were both well calibrated and had a high net clinical benefit.

**Conclusion:**

Our findings suggest that the prevalence of motor complications during long‐term LD treatment is relatively high among PD patients in China, with WO occurring more commonly than dyskinesia. Younger age at PD onset, higher LEDD, more severe disease, and akinetic‐rigid subtype are key predictors of motor complications. The predictive models developed in this study could serve as a potential tool to assist clinicians in identifying patients at higher risk for WO and dyskinesia, and may support personalized treatment optimization.

## Graphical abstract

The study demonstrates that younger age at PD onset, higher LEDD and more severe disease are independent risk factors for WO, whereas younger age at PD onset, akinetic‐rigid subtype and higher LEDD are independent risk factors for dyskinesia. The predictive models developed have high clinical predictive value and may serve as a robust tool for early identification of high‐risk patients.

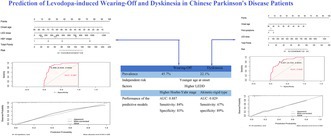

## 1 Introduction

Parkinson's disease (PD) is one of the most common neurodegenerative disorders, which primarily involves nigrostriatal dopaminergic neurons [1]. PD patients manifest a group of clinical syndromes combining both motor and non‐motor symptoms [1, 2]. Currently, levodopa (LD) is recognized as the most widely used and effective drug for the treatment of PD [3, 4]. However, as the disease progresses, LD‐associated motor complications increase, such as the end‐of‐dose wearing‐off (WO) phenomenon and levodopa‐induced dyskinesia (LID) [5, 6, 7]. This not only poses significant challenges to neurologists but also seriously affects the quality of life of PD patients [8]. Therefore, it is important to identify patients at high risk of developing motor complications, thus providing a reliable theoretical basis for the formulation of clinical treatment protocols.

PD patients often begin to develop motor complications after 1–5 years of LD treatment, and a small number of patients develop motor complications within 1 year after LD treatment [9, 10]. Several observational studies have shown that in Western countries, approximately 40% of patients treated with LD for 4–6 years develop motor complications [10, 11], the prevalence of WO ranges from 22% to 64%, and that of LID from 26% to 44% [6, 12, 13]. The incidence of motor complications increases with the duration of disease [8, 14, 15]. More than half of patients develop motor complications within 5 years of diagnosis [16]. At the end of the first decade, up to 90% of patients may develop LID, and 60% may develop WO [17]. At the end of the second decade, almost 100% of patients taking more than 300 mg of LD per day develop motor complications [18]. Limited epidemiological data are available on PD in China. Data reported from Hong Kong [19] and mainland China [20] have shown significant heterogeneity. It is critical to explore the true nature of LD‐induced motor complications in the Chinese context. To fill the research gap in the literature, the study aimed to (1) evaluate the prevalence of motor complications in LD‐treated PD patients in China and (2) analyze the influence of demographic and clinical factors on motor complications. Through this investigation, we hope to provide more effective treatment strategies for PD patients in China, especially for the management of motor complications, and provide more data on PD in Asia globally.

## 2 Materials and Methods

### 2.1 Study Population and Survey Design

This is a cross‐sectional study. The study was approved by the Medical Ethics Committee of the Aerospace Center Hospital (Approval No. 20190614‐SF‐06). All patients gave their written informed consent to participate in the study.

Consecutive PD patients attending the PD‐specialized outpatient clinic of Aerospace Center Hospital from September 2019 to June 2021 were recruited. At each visit, eligible PD patients were preliminarily screened by experienced neurologists specializing in PD. All patients were asked to fill out a 9‐item wearing‐off questionnaire (WOQ‐9) and a basic information registration form. For patients who had at least one positive response of WOQ‐9 (WOQ‐9 ≥ 1), a detailed case report form was filled in. The final diagnosis of WO and dyskinesia was made by a movement disorders specialist. WO is defined as the shortening of benefit from each LD dose to less than 4 h [21]. Dyskinesia is defined as the occurrence of choreiform movements involving the extremities, neck, trunk, or occasionally the face, observed during the “on” period in patients exhibiting significant improvement in PD symptoms [21].

The inclusion criteria included: an age of ≥ 18 years; patients who met the International Parkinson's Disease and Movement Disorder Society 2015 Diagnostic Criteria for PD [22]; and patients who were willing to participate and provided written informed consent.

The exclusion criteria included: patients who had intracranial organic diseases or atypical parkinsonism (multiple system atrophy, progressive supranuclear palsy, and dementia with Lewy Bodies), secondary parkinsonism due to vascular causes, toxins, trauma, and metabolic causes; patients who had psychiatric, consciousness, or cognitive disorders; and patients who took antipsychotic drugs.

According to the presence and absence of WO and dyskinesia, patients were divided into groups with and without WO, as well as groups with and without dyskinesia.

### 2.2 Diagnostic Criteria

Eligible PD patients were assessed for WO by the WOQ‐9 [23]. The WOQ‐19 consists of motor and non‐motor symptoms. For each item, patients reported whether a symptom was present and whether that symptom had improved with anti‐PD medication. If both were positive, a score of 1 was obtained. The possible presence of WO was defined by a WOQ‐9 score of at least 1 point.

Dyskinesia was assessed using the Unified Parkinson's Disease Rating Scale part IV (UPDRS‐IV) [24]. UPDRS‐IV is made up of four questions, including the duration and severity of movement disorders. Higher scores indicate more severe movement disorders.

### 2.3 General Demographic Characteristics

Baseline and clinical data were collected from all enrolled PD patients, which included demographic characteristics (such as age, gender, smoking, alcohol consumption, height, weight and concomitant diseases) and clinical characteristics (onset age, disease duration, type of first symptom, duration of LD therapy, and history of using medications). The LD dose and levodopa‐equivalent daily dose (LEDD) were calculated according to the methods described in a previous study [25].

### 2.4 Scale Evaluation

The Montreal Cognitive Assessment (MoCA) and Mini‐Mental State Examination (MMSE) scales were used to assess the cognitive functions in PD patients. Hamilton Anxiety Rating Scale (HAMA) and Hamilton Depression Rating Scale (HAMD) were used to assess patients' mood states. The Hoehn‐Yahr (H‐Y) scale was used for assessing the severity of PD, with higher scores indicating more severe disease. H‐Y stage ≤ 2.5 is defined as early‐stage PD, and H‐Y stage > 2.5 is defined as middle‐late stage PD [22]. All patients were assessed with Unified Parkinson's Disease Rating Scale part III (UPDRS III), with higher scores indicating poorer motor function in PD patients.

### 2.5 Statistical Analysis

Continuous variables were tested for normality using the Shapiro–Wilk test to assess whether they followed a normal distribution. If the data did not exhibit a normal distribution, they were expressed as median (interquartile range, IQR) and analyzed using a non‐parametric test (Mann–Whitney *U* test). Categorical variables were described as frequency (percentage), and group differences were assessed using the chi‐square test. Univariate and multivariate logistic regression analyses were used to predict the risk factors of WO and dyskinesia. Predictive models for WO and dyskinesia were constructed based on the results of multivariate analysis. The model discrimination was evaluated using the area under the receiver operating characteristic curve (AUC, 95% CI). The calibration of the models was assessed using the Hosmer‐Lemeshow test and calibration curve analysis. Decision curve analysis was performed to quantify clinical utility and determine the net benefit of the predictive models. All analyses were performed using R version 4.2.1, with a two‐sided significance at *p* < 0.05.

## 3 Results

### 3.1 General Demographic Characteristics

**FIGURE 1:**
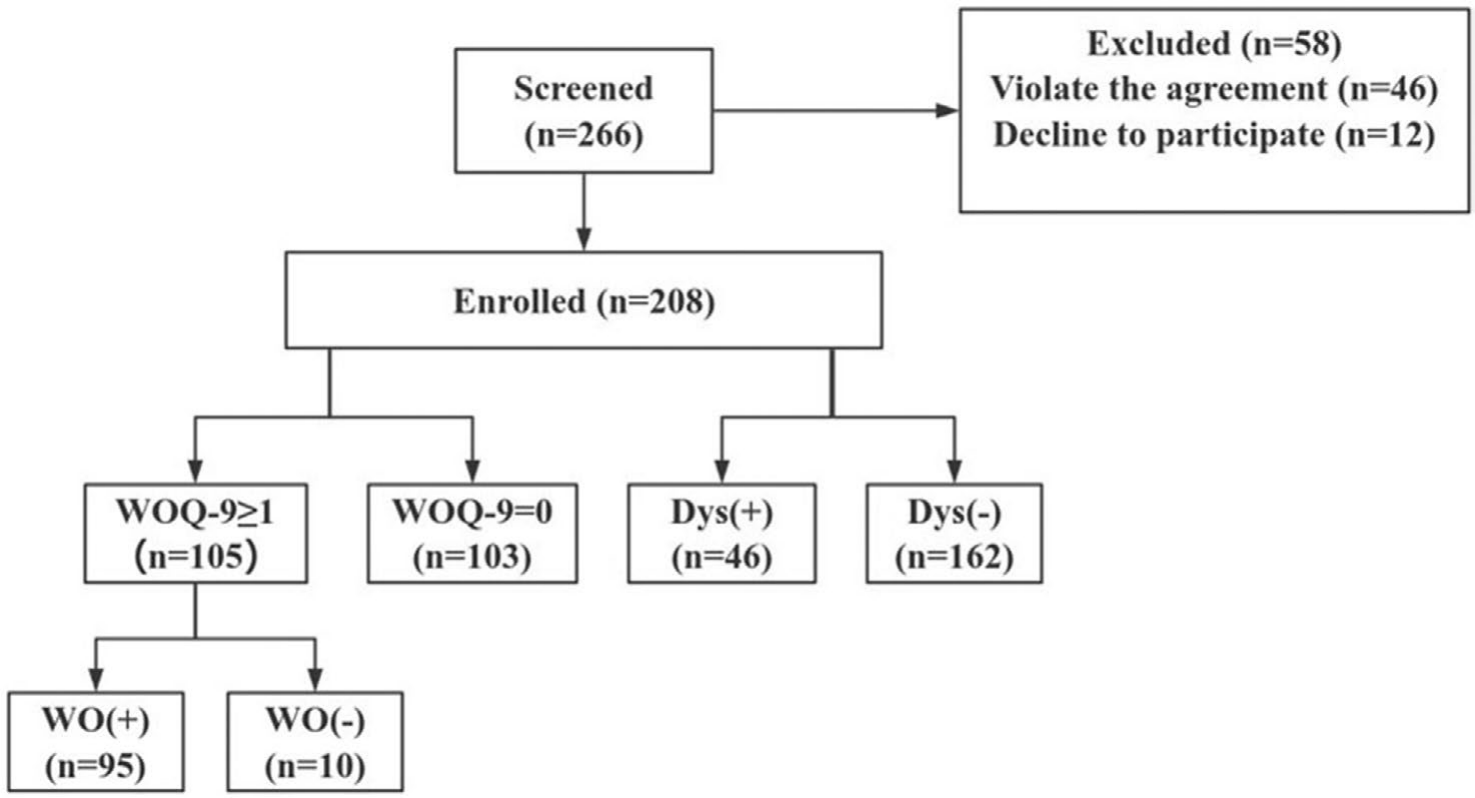
Flow chat of screening and enrollment of PD patients with WO and dyskinesia through the survey. Dys, Dyskinesia; WO, Wearing‐off; WOQ‐9, 9‐item wearing‐off questionnaire.

The process of participant screening and enrollment is depicted in Figure 1. Of the 266 patients screened, 58 patients were excluded, including 46 who were diagnosed with other diseases and 12 patients declined enrollment. A total of 208 patients were ultimately included, of which 115 (55.3%) were male. The median age of patients was 67 years (range 60–74), and the median age at disease onset was 60 years (range 51–70). The median disease duration was 5.0 years (range 3.0–9.0), and the median LEDD was 475 mg/day (range 344–675).

### 3.2 Occurrence of Motor Complications

Of the 208 PD patients included in the study, 96 (46.2%) patients had motor complications, with the occurrence of WO phenomenon in 95 (45.7%) patients, dyskinesia in 46 (22.1%) patients, and the simultaneous occurrence of WO and dyskinesia in 45 (21.6%) patients.

### 3.3 Baseline Characteristics of PD Patients With and Without Motor Complications

Table 1 summarizes the baseline characteristics of PD patients with and without WO and dyskinesia. Compared with patients without motor complications, patients with motor complications were younger, had a younger age at PD onset, longer disease duration, longer duration of LD therapy, higher LEDD, and more severe disease. Additionally, patients with motor complications had a higher risk of developing dysphagia, dysarthria, and falls, and received deep brain stimulation more frequently than those without motor complications.

**TABLE 1:** Baseline characteristics of PD patients with and without WO and dyskinesia.

Variables	WO		*p*	Dyskinesia		*p*
	With (*n* = 95)	Without (*n* = 113)		With (*n* = 46)	Without (*n* = 162)	
Age, years	64.0 [58.0; 71.0]	68.0 (61.0; 76.0)		64.0 [56.3, 71.0]	68.0 [61.0, 74.8]	
Onset age, years	55.0 [48.0;61.0]	65.0 [55.0; 73.0]		51.0 [46.0; 59.8]	61.0 [54.0; 71.5]	
Gender (Female, %)	41 (43.2%)	52 (46.0%)	0.785	22 (47.8%)	71 (43.8%)	0.754
BMI, kg/m^2^	23.4 [21.1; 25.4]	24.0 [22.2; 25.4]	0.183	22.7 [20.6, 24.7]	23.9 [22.1, 25.4]	
First symptoms (*n*, %)			0.187			
Tremor‐dominant subtype	40 (42.1%)	62 (54.9%)		15 (32.6%)	87 (53.7%)	
Akinetic‐rigid subtype	51 (53.7%)	47 (41.6%)		29 (63.0%)	69 (42.6%)	
Mixed subtype	4 (4.21%)	4 (3.54%)		2 (4.35%)	6 (3.70%)	
Disease duration, years	8.00 [6.00;12.0]	3.00 [1.50; 5.00]		9.50 [6.62; 13.0]	4.25 [2.00; 7.00]	
Duration of LD therapy, years	7.50 [5.50; 11.0]	2.50 [1.00; 4.25]		8.50 [5.62; 12.4]	3.50 [1.50; 6.50]	
LEDD, mg/d	650 [500; 790]	388 [300; 475]		675 [521; 854]	419 [303; 600]	
≤ 400 mg/d (*n*, %)	51 (53.68)	89 (78.76)		24 (52.17)	116 (71.60)	
400–600 mg/d (*n*, %)	31 (32.63)	22 (19.47)		14 (30.43)	39 (24.07)	
> 600 mg/d (*n*, %)	13 (13.68)	2 (1.77)		8 (17.39)	7 (4.32)	
Hoehn‐Yahr stage	2.50 [2.50; 3.00]	2.50 [2.00; 2.50]		2.50 [2.12; 3.00]	2.50 [2.00; 3.00]	
1–2.5 (*n*, %)	15 (15.79)	65 (57.52)		5 (10.87)	75 (46.30)	
3 (*n*, %)	25 (26.32)	36 (31.86)		12 (26.09)	49 (30.25)	
4–5 (*n*, %)	55 (57.89)	12 (10.62)		29 (63.04)	38 (23.46)	
UPDRS‐III	36.0 [26.5;50.0]	29.0 [20.0;39.0]		33.0 [24.5; 45.8]	32.0 [24.0; 42.0]	0.663
Smoking (*n*, %)	77 (81.1%)	97 (85.8%)	0.458	41 (89.1%)	133 (82.1%)	0.362
Alcohol consumption (*n*, %)	75 (78.9%)	91 (80.5%)	0.912	38 (82.6%)	128 (79.0%)	0.743
Hypertension (*n*, %)	18 (18.9%)	32 (28.3%)	0.158	10 (21.7%)	40 (24.7%)	0.827
T2DM (*n*, %)	5 (5.26%)	14 (12.4%)	0.125	1 (2.17%)	18 (11.1%)	0.081
Anxiety (*n*, %)	32 (33.7%)	29 (25.7%)	0.266	13 (28.3%)	48 (29.6%)	1.000
Depression (*n*, %)	62 (65.3%)	47 (41.6%)		29 (63.0%)	80 (49.4%)	0.142
MMSE, score	24.8 (4.64)	26.7 (3.81)		26.0 [23.0; 29.0]	27.0 [24.0; 29.0]	0.248
MOCA, score	21.6 (5.67)	23.5 (4.75)		24.0 [20.2; 26.0]	24.0 [19.0; 27.0]	0.678
Bladder dysfunction (*n*, %)	18 (18.9%)	6 (5.31%)		5 (10.9%)	19 (11.7%)	1.000
Dysphagia (*n*, %)	10 (10.5%)	1 (0.88%)		6 (13.0%)	5 (3.09%)	
Dysarthria (*n*, %)	15 (15.8%)	6 (5.31%)		8 (17.4%)	13 (8.02%)	0.092
Falls (*n*, %)	28 (29.5%)	14 (12.4%)		16 (34.8%)	26 (16.0%)	
DBS treatment (*n*, %)	16 (16.8%)	1 (0.88%)		14 (30.4%)	3 (1.85%)	

Abbreviations: BMI, body mass index; DBS, deep brain stimulation; LEDD, levodopa equivalent daily dose; MMSE, Mini‐Mental State Examination; MOCA, Montreal Cognitive Assessment; PD, Parkinson's disease; T2DM, type 2 diabetes mellitus; UPDRS‐III, Unified Parkinson's Disease Rating Scale part III; WO, wearing‐off.

* *p*‐value < 0.05.

Patients with WO had a higher UPDRS‐III score, lower MMSE and MOCA scores, with depression and bladder dysfunction occurring more commonly than patients without WO. Patients with dyskinesia had a lower body mass index than those without dyskinesia. These two groups also differed significantly in terms of their first symptoms, with akinetic‐rigid type occurring more frequently in patients with dyskinesia.

### 3.4 Risk Factors Associated With the Occurrence of WO and Construction of Predictive Model

Table 2 shows the univariate and multivariate logistic regression analyses of the risk factors associated with the occurrence of WO. Univariate analysis showed that age (OR 0.97, 95% CI 0.95–0.99, *p* = 0.027), onset age (OR 0.94, 95% CI 0.91–0.96, *p* < 0.001), disease duration (OR 1.55, 95% CI 1.38–1.77, *p* < 0.001), duration of LD therapy (OR 1.56, 95% CI 1.38–1.78, *p* < 0.001), LEDD (OR 1.01, 95% CI 1.00–1.01, *p* < 0.001), H‐Y stage (OR 3.41, 95% CI 2.04–5.18, *p* < 0.001), UPDRS‐III score (OR 1.03, 95% CI 1.01–1.05, *p* < 0.001), depression (OR 2.64, 95% CI 1.51–4.67, *p* = 0.001), MMSE (OR 0.90, 95% CI 0.84–0.96, *p* = 0.003) and MOCA score (OR 0.94, 95% CI 0.89–0.99, *p* = 0.019) were significantly associated with an increased risk of WO.

**TABLE 2:** Univariable and multivariable logistic regression analyses of risk factors associated with WO in patients with PD.

Variables	Univariable		Multivariable	
	OR (95% CI)	*p*	OR (95% CI)	*p*
Age	0.97 (0.95 ~ 0.99)	**0.027**		
Onset age	0.94 (0.91–0.96)	**< 0.001**	**0.92 (0.89–0.95)**	**< 0.001**
Gender	0.89 (0.51–1.54)	0.679		
BMI	0.95 (0.86 ~ 1.04)	0.269		
First symptoms				
Tremor‐dominant subtype	Reference			
Akinetic‐rigid subtype	1.68 (0.96–2.96)	0.069		
Mixed subtype	1.55 (0.35–6.90)	0.551		
Disease duration	1.55 (1.38–1.77)	**< 0.001**		
Duration of LD therapy	1.56 (1.38–1.78)	**< 0.001**		
LEDD	1.01 (1.00–1.01)	**< 0.001**	**1.00 (1.00–1.01)**	**< 0.001**
Hoehn‐Yahr stage	3.14 (2.04–5.18)	**< 0.001**	**3.41 (2.00–5.83)**	**< 0.001**
UPDRS‐III	1.03 (1.01–1.05)	**< 0.001**		
Smoking	0.71 (0.33–1.48)	0.354		
Alcohol consumption	0.91 (0.46–1.80)	0.777		
Hypertension	0.59 (0.30–1.13)	0.117		
T2DM	0.39 (0.12–1.07)	0.084		
Anxiety	1.47 (0.81–2.69)	0.207		
Depression	2.64 (1.51–4.67)	**0.001**		
MMSE	0.90 (0.84–0.96)	**0.003**		
MOCA	0.94 (0.89–0.99)	**0.019**		

Abbreviations: BMI, body mass index; LD, levodopa; LEDD, levodopa equivalent daily dose; MMSE, Mini‐Mental State Examination; MOCA, Montreal Cognitive Assessment; PD, Parkinson's disease; T2DM, type 2 diabetes mellitus; UPDRS‐III, Unified Parkinson's Disease Rating Scale part III; WO, wearing‐off.

Multivariate logistic regression analysis showed that onset age (OR 0.92, 95% CI 0.89–0.95, *p* < 0.001), LEDD (OR 1.00, 95% CI 1.00–1.01, *p* < 0.001) and H‐Y stage (OR 3.41, 95% CI 2.00–5.83, *p* < 0.001) were independent risk factors for the occurrence of WO.

**FIGURE 2:**
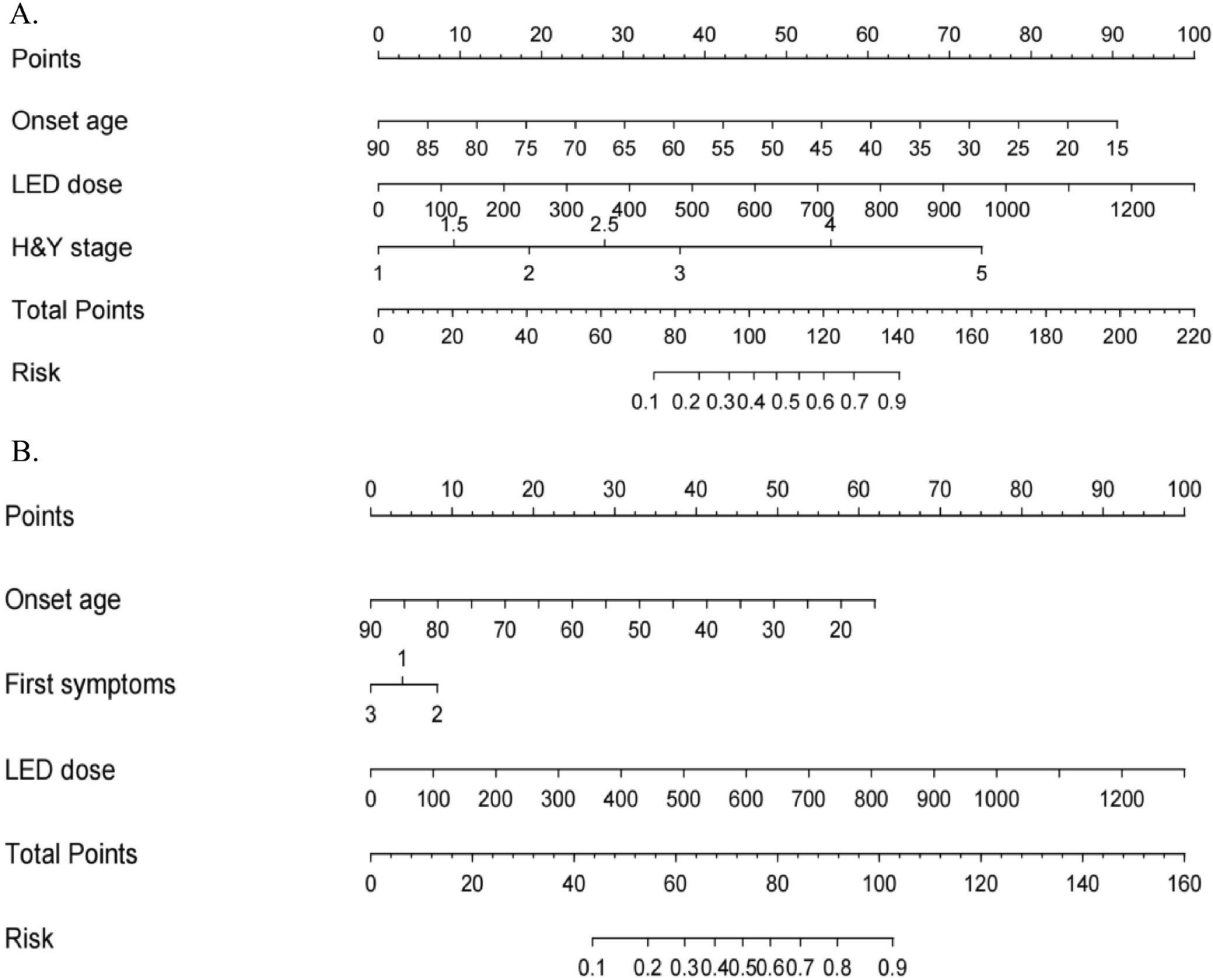
Nomograms for predicting wearing‐off (WO) and dyskinesia during long‐term levodopa treatment in patients with Parkinson's disease. (A) Nomogram constructed based on onset age, levodopa equivalent daily dose (LEDD), and Hoehn‐Yahr (H‐Y) stage for predicting the risk of WO in patients with Parkinson's disease. Each variable contributes to a corresponding score, and the total score is calculated by summing the scores of all variables, which uses to determine the risk of WO. (B) Nomogram constructed based on onset age, first symptoms, and LEDD for predicting the risk of dyskinesia in patients with Parkinson's disease. Each variable contributes to a corresponding score, and the total score is calculated by summing the scores of all variables, which uses to determine the risk of dyskinesia.

**FIGURE 3:**
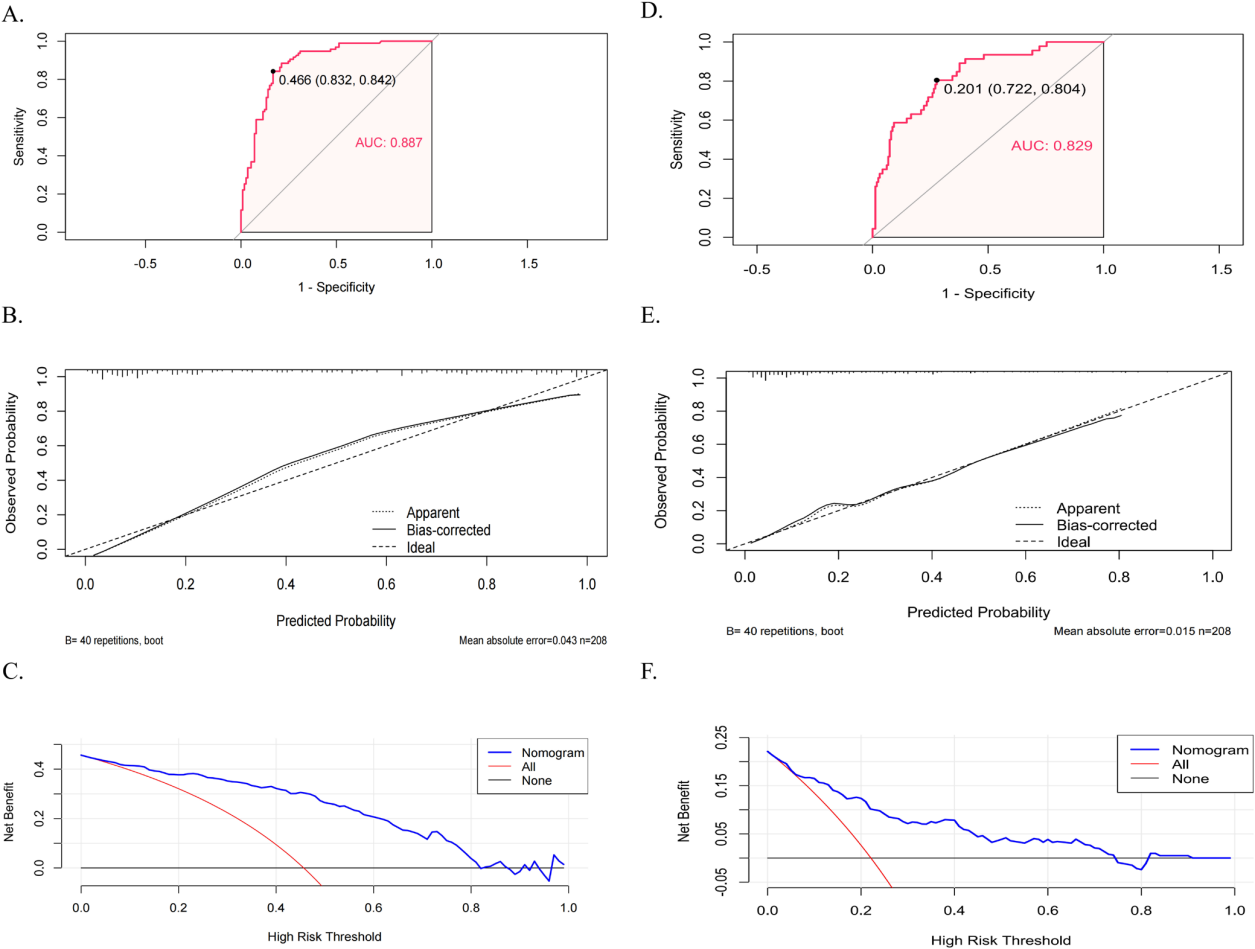
Performance and clinical utility of the predictive models for wearing‐off (WO) and dyskinesia. (A) Receiver operating characteristic (ROC) curve for the predictive model of WO, showing an AUC of 0.887 (95% CI 0.832–0.842). (B) Calibration curve for the predictive model of WO, showing the agreement between the predicted and observed probabilities, with bias‐corrected and ideal curves for comparison. (C) Decision curve analysis (DCA) for the predictive model of WO, demonstrating the net clinical benefit at varying risk thresholds. (D) ROC curve for the predictive model of dyskinesia, with an AUC of 0.829 (95% CI 0.722–0.804). (E) Calibration curve for the predictive model of dyskinesia, displaying the comparison between the predicted and observed probabilities, along with bias‐corrected and ideal curves. (F) DCA for the predictive model of dyskinesia, illustrating the net clinical benefit across different high‐risk thresholds.

Based on the results of multivariate logistic regression analysis, a nomogram was constructed using the three variables to predict the risk of WO in PD patients (Figure 2A). The predictive model demonstrated high diagnostic efficacy with an AUC of 0.887 (95% CI 0.842–0.932), a sensitivity of 84%, and a specificity of 83% (Figure 3A, Table 4). The calibration curve and DCA showed that the model was well calibrated and had a high net clinical benefit (Figure 3B,C).

### 3.5 Risk Factors Associated With the Development of Dyskinesia and Construction of Predictive Model

Table 3 shows the univariate and multivariate logistic regression analyses of the risk factors for dyskinesia. Univariate analysis showed that age (OR 0.96 95% CI 0.94–0.99, *p* = 0.008), onset age (OR 0.93, 95% CI 0.91–0.96, *p* < 0.001), akinetic‐rigid subtype as the first symptom (OR 2.44, 95% CI 1.21–4.90, *p* = 0.012), disease duration (OR 1.26, and 95% CI 1.16–1.37, *p* < 0.001), duration of LD therapy (OR 1.27, 95% CI 1.17–1.38, *p* < 0.001), LEDD (OR 1.01, 95% CI 1.01–1.01, *p* < 0.001), and H‐Y stage (OR 1.94, 95% CI 1.30–2.91, *p* = 0.001) were risk factors for the development of dyskinesia.

**TABLE 3:** Univariable and multivariable logistic regression analyses of risk factors associated with dyskinesia in patients with PD.

Variables	Univariable		Multivariable	
	OR (95% CI)	*p*	OR (95% CI)	*p*
Age	0.96 (0.94 ~ 0.99)	**0.008**		
Onset age	0.93 (0.91 ~ 0.96)	**< 0.001**	0.94 (0.91 ~ 0.97)	**< 0.001**
Gender	1.17 (0.61 ~ 2.27)	0.630		
BMI	0.90 (0.80 ~ 1.01)	0.085		
First symptoms				
Tremor‐dominant subtype	Reference			
Akinetic‐rigid subtype	2.44 (1.21 ~ 4.90)	**0.012**	2.42 (1.07 ~ 5.47)	**0.034**
Mixed subtype	1.93 (0.36 ~ 10.49)	0.445		
Disease duration	1.26 (1.16 ~ 1.37)	**< 0.001**		
Duration of LD therapy	1.27 (1.17 ~ 1.38)	**< 0.001**		
LEDD	1.01 (1.01 ~ 1.01)	**< 0.001**	1.01 (1.01 ~ 1.01)	**< 0.001**
Hoehn‐Yahr stage	1.94 (1.30 ~ 2.91)	**0.001**		
UPDRS‐III	1.01 (0.99 ~ 1.03)	0.392		
Smoking	1.79 (0.65 ~ 4.92)	0.260		
Alcohol consumption	1.26 (0.54 ~ 2.96)	0.592		
Hypertension	0.85 (0.39 ~ 1.86)	0.679		
T2DM	0.18 (0.02 ~ 1.37)	0.097		
Anxiety	0.18 (0.02 ~ 1.37)	0.097		
Depression	1.75 (0.89 ~ 3.43)	0.104		
MMSE	0.95 (0.89 ~ 1.03)	0.211		
MOCA	0.98 (0.93 ~ 1.05)	0.632		

Abbreviations: BMI, body mass index; LD, levodopa; LEDD, levodopa equivalent daily dose; MMSE, Mini‐Mental State Examination; MOCA, Montreal Cognitive Assessment; PD, Parkinson's disease; T2DM, type 2 diabetes mellitus; UPDRS‐III, Unified Parkinson's Disease Rating Scale part III; WO, wearing‐off.

Multivariate logistic regression analysis showed that onset age (OR 0.94, 95% CI 0.91–0.97, *p* < 0.001), akinetic‐rigid subtype (OR 2.42, 95% CI 1.07–5.47, *p* = 0.034), and LEDD (OR 1.01, 95% CI 1.01–1.01, *p* < 0.001) were independently associated with the development of dyskinesia. Similarly, a nomogram was constructed using the three variables to predict the risk of dyskinesia in PD patients (Figure 2B). The predictive model yielded an AUC value of 0.829 (95% CI 0.767–0.897), with a sensitivity of 67% and a specificity of 89% (Figure 3D, Table 4). The calibration curve and DCA showed that the model had good calibration and high net clinical benefit in predicting the risk of dyskinesia (Figure 3E,F).

**TABLE 4:** The diagnostic performance of two models for predicting WO and dyskinesia in patients with PD.

Variables	WO	Dyskinesia
AUC (95% CI)	0.887 (0.842–0.932)	0.829 (0.767–0.897)
Sensitivity (95% CI)	83.2 (76.3–90.1)	66.7 (59.4–73.9)
Specificity (95% CI)	84.2 (76.9–91.5)	89.1 (80.1–98.1)
PPV (95% CI)	86.2 (79.8–92.7)	95.6 (91.8–99.4)
NPV (95% CI)	80.8 (73.1–88.6)	43.2 (33.2–53.1)

Abbreviations: AUC, area under the curve; NPV, negative predictive value; PD, Parkinson's disease; PPV, positive predictive value; WO, wearing‐off.

## 4 Discussion

Although motor complications associated with LD therapy in PD have been widely reported [6, 8, 9, 10], studies on this issue in PD patients in China are lacking. This cross‐sectional study included 208 Chinese patients with idiopathic PD and found that the prevalence of motor complications was 46.2% (96/208), with the rate of WO being higher than dyskinesia (45.7% [95/208] vs. 22.1% [46/208]). This finding is consistent with most international studies [16, 26]. Notably, it has been shown that the prevalence of WO in China (45.7%) was higher than that in Europe and the United States (20.1%–43.9%) [27, 28], but significantly lower than that in Japan (56.1%) [6]. The prevalence of LID in China (22.1%) was significantly lower than the findings from the Parkinsonism Incidence in North‐East Scotland (PINE) study (37%) [13], and international long‐term follow‐up study (40%–50% by 5 years of treatment and > 90% by 10 years) [29, 30], which is similar to the findings of Kadastik‐Eerme et al. (21%) [28]. The results of this study also showed that PD patients with WO and dyskinesia had significantly longer duration of LD therapy when compared to patients without WO and dyskinesia (7.5 years vs. 2.5 years; 8.5 years vs. 3.5 years, respectively, all *p* < 0.001). The results corroborate with the risk factors for motor complications (younger age at diagnosis, higher cumulative LD dose, female sex) identified in the PINE study [13]. A multicenter study conducted in Shanghai, China reported a prevalence of 46.5% for WO, which is similar to our study, but the study reported a lower prevalence of LID (11.4%). The regional differences in the prevalence rates may be due to factors such as disease duration, disease severity, and medication strategies (e.g., generally low LD dosage and late LD administration). In addition, differences in study design (such as sample characteristics, follow‐up time), racial/ethnic differences in drug metabolism and lifestyle, and complex interactions among risk factors [12, 31] can also lead to heterogeneity across studies. Meanwhile, it should be noted that in this study, alternative diagnoses were identified in 46 of these 266 patients; this underscores the challenges in accurate diagnosis of PD, particularly in regions with limited access to movement disorders specialists, thus emphasizing the ongoing need for diagnostic refinement.

The mechanism by which motor complications occur in PD patients involves the combined action of multiple factors: (1) loss of nigrostriatal dopamine terminals due to disease progression and fluctuating levels of LD due to central and peripheral pharmacokinetic mechanisms together constitute the pathophysiological basis of motor complications in PD [32]. (2) Genetic factors, the use of other dopaminergic therapies, psychiatric comorbidities, and lifestyle factors may also influence the development of motor complications in PD. Genetic factors are more commonly observed in LD‐induced dyskinesias, such as LRRK2, PARK2 (parkin), PARK6 (pink‐1), and PARK7 (DJ‐1) [33, 34, 35]. Polymorphisms of the dopamine D2 receptor and transporter gene, as well as the TaqIA polymorphism located in the gene encoding the D2 receptor, have been shown to be associated with the risk of developing dyskinesias in PD [36, 37]. Although among anti‐PD medications, LD is most closely associated with the occurrence of motor complications in PD, other drugs such as DA agonists, MAO‐B inhibitors, and COMT inhibitors may also influence the development of motor complications to varying degrees. Additionally, studies have shown that baseline non‐motor symptoms, such as lower BMI, educational level, psychiatric comorbidities (apathy, depression and anxiety) [38, 39], can significantly impact the development and progression of motor complications. (3) Studies in western countries showed that clinical parameters including younger age at onset [16, 40], female gender [13], initial treatment with LD [14] and a higher score on the UPDRS Part II [13, 40] were associated with increased risk of developing motor complications. The findings of this study confirm this complexity, showing that PD patients who developed motor complications had a younger age, younger onset age, longer disease duration, longer duration of LD therapy, higher LEDD, and greater disease severity. These characteristics are highly consistent with the results of studies by Warren Olanow et al. [40] and Kadastik‐Eerme et al. [28] showing that younger age at onset of the disease, shorter time to LD initiation, higher LEDD, and akinetic‐rigid dominant phenotype of PD were significantly associated with the occurrence of motor complications. These findings further confirm the critical role of disease progression and long‐term LD exposure in the occurrence of motor complications.

In this study, we systematically analyzed the risk factors of WO, and found that younger age at onset, longer disease duration, longer duration of LD therapy, higher LEDD, greater disease severity, and higher UPDRS‐III score significantly increased the risk of WO. In this study, further multifactorial logistic regression analysis demonstrated that H‐Y stage was the strongest predictor, with the highest OR (3.41), suggesting that overall disease progression is a core driver for the occurrence of both motor and non‐motor WO symptoms [40]. Furthermore, younger age at onset (OR 0.92) and higher LEDD (OR 1.00) were also independent risk factors for WO, indicating that younger patients and those treated with high‐dose LD need to be more closely monitored. The predictive model constructed based on these three key variables demonstrated excellent diagnostic efficacy with an AUC of 0.887, providing a quantitative tool for clinical decision‐making. These findings not only support that WO is a result of the combined effect of disease progression and long‐term LD treatment, but also emphasize the need to comprehensively evaluate the disease stage, patients' age and LD dose when formulating appropriate treatment strategies. Future studies should focus on exploring the interaction between disease progression and LD treatment in order to optimize treatment timing and protocol.

The results of this study showed that in comparison of patients without dyskinesia, patients with dyskinesia had younger age at PD onset, longer disease duration, higher LD dose, and longer duration of LD therapy, and more severe disease. These findings are consistent with previous studies [8, 12]. Multifactorial analysis further showed that younger age at PD onset, the presence of akinesia and rigidity as first symptoms and higher LEDD were independent risk factors for the development of dyskinesia, which are consistent with previous findings [41, 42]. It is worth noting that young patients may be more inclined to receive high‐dose LD therapy due to social functioning needs, this can lead to higher LEDD intake and finally, an increased risk of LID. Dose–response relationship studies have shown a positive correlation between LD dose and prevalence of LID. For example, the Earlier versus Later Levodopa Therapy in Parkinson's Disease (ELLDOPA) study reported that the prevalence rates of LID were 3.4%, 16.5% and 16.5%, respectively, after treatment with LD at a dose of 150, 300 and 600 mg per day [43]. And the Stalevo Reduction in Dyskinesia Evaluation in Parkinson's Disease (STRIDE‐PD) study demonstrated that even when combined with entacapone, LD 400 mg/day still significantly increased the risk of LID. In this study, the mean LEDD dose was 475 mg/day, which is consistent with the findings mentioned above. These findings once again emphasize the importance of the treatment principle of “minimum effective dose”, especially in patients with early‐onset PD. In terms of treatment for dyskinesia, the available evidence supports that deep brain stimulation surgery targeting the globus pallidus internus can serve as an effective intervention to reduce dyskinesia, while Amantadine extended release is the preferred medication for the treatment of dyskinesia [44].

Another major finding of this study was that PD patients with the akinetic‐rigid subtype as the first symptom had a higher risk of developing dyskinesia. This is consistent with the results of a previous study showing that patients with the tremor‐dominant subtype had a low risk of developing dyskinesia [28]. An observational study on 144 LD‐treated patients showed that patients with the akinetic‐rigid dominant subtype had a significantly higher risk of developing LID compared to those with the tremor‐dominant subtype (69% vs. 29%) [45]. A previous study compared the differences in striatal dopamine transporter protein binding and the extent of caudate dopamine terminal loss between patients with akinetic‐rigid and tremor‐dominant subtypes and found that dopaminergic function is relatively well spared in patients with tremor‐dominant PD compared to those with akinetic‐rigid PD [46]. In this study, a predictive model for dyskinesia constructed using the three variables, including the akinetic‐rigid subtype (2.42), age at PD onset (OR 0.94), and LEDD (OR 1.01), yielded an AUC of 0.829 (95% CI 0.767–0.897). The akinetic‐rigid subtype was the strongest predictor of dyskinesia, with the highest OR (2.42). Therefore, achieving effective treatment in patients with the akinetic‐rigid subtype as the predominant symptom is more challenging.

The study has several limitations. First, this is a cross‐sectional study, which cannot delineate the causal relationships between the identified risk factors and the development of motor complications. The fact that the data was collected from a single center may limit the generalizability of the results. Further large‐scale, multi‐center longitudinal studies are needed to empirically substantiate their causal relationships and confirm our findings. Second, the sample size of 208 patients, while adequate for initial findings, is relatively small compared to the large number of individuals affected by PD in China. Therefore, further studies in the future using a larger sample size or more representative samples would be required to validate the present results. Third, the model was only internally validated; an external validation study is needed to evaluate the generalizability of the predictive model. The model only incorporated clinical parameters related to motor complications, including medicine, disease, and patient factors; further studies are warranted to evaluate the role of genetic factors in predicting motor complications. Furthermore, in this study, data collection relied partially on self‐reported measures, which may introduce recall and subjective biases. In future studies, it is necessary to incorporate objective measures, such as wearable sensors or clinician‐rated scales, to improve the accuracy and reliability of the data.

## 5 Conclusions

Through a cross‐sectional analysis of 208 PD patients in China, the study revealed a high prevalence of motor complications during long‐term LD treatment in Chinese PD patients (46.2%), with WO occurring more commonly than dyskinesia (45.7% vs. 22.1%). The study demonstrated that younger age at PD onset, higher LEDD, and more severe disease are independent risk factors for WO, whereas younger age at PD onset, akinetic‐rigid subtype, and higher LEDD are independent risk factors for dyskinesia. Two predictive models for WO and dyskinesia constructed based on these risk factors yielded an AUC of 0.887 and 0.829, respectively, which had high clinical predictive value and may provide a basis for personalized treatment. The study highlights the importance of early identification of high‐risk patients and optimization of LD dosage and provides critical data for managing motor complications in PD patients in Asia.

## Author Contributions

Study concept and design: J.D., Z.W. Acquisition of data: J.Z., Y.G., C.S., J.C., Y.W., J.C., S.M., P.W., J.L. Analysis and interpretation of data: J.Z., Y.G., C.S., J.C., Y.W., J.C., S.M., P.W., J.L. Drafting of the manuscript: J.Z. Critical revision of the manuscript for important intellectual content: J.D., Z.W. Study supervision: J.Z., J.D., Z.W.

## Conflicts of Interest

The authors declare no conflicts of interest.

## Data Availability

The data and code that underpin the findings of this study are available from the corresponding authors, upon reasonable request.
